# Recent advances in imaging subcellular processes

**DOI:** 10.12688/f1000research.8399.1

**Published:** 2016-06-30

**Authors:** Kenneth A. Myers, Christopher Janetopoulos

**Affiliations:** 1 Department of Biological Sciences, University of the Sciences in Philadelphia, Philadelphia, PA, USA

**Keywords:** imaging subcellular processes, microscope, cell biology, Stimulated emission-depletion microscopy, STED, microscopy techniques

## Abstract

Cell biology came about with the ability to first visualize cells. As microscopy techniques advanced, the early microscopists became the first cell biologists to observe the inner workings and subcellular structures that control life. This ability to see organelles within a cell provided scientists with the first understanding of how cells function. The visualization of the dynamic architecture of subcellular structures now often drives questions as researchers seek to understand the intricacies of the cell. With the advent of fluorescent labeling techniques, better and new optical techniques, and more sensitive and faster cameras, a whole array of questions can now be asked. There has been an explosion of new light microscopic techniques, and the race is on to build better and more powerful imaging systems so that we can further our understanding of the spatial and temporal mechanisms controlling molecular cell biology.

## Introduction

Optical microscopy is a diffraction-limited imaging methodology. As such, the use of light to generate a microscopic image is inherently bound by the wavelength(s) of light used to generate a diffraction pattern and the aperture of the objective lens oriented to collect the diffracted light source. These limitations were first described mathematically by Ernst Karl Abbe as a lateral resolution limit equivalent to the wavelength of light divided by two times the numerical aperture of the objective lens (d = λ/2NA) ^1^. With this fundamental resolution limit established and verified experimentally, a century of light microscopy working at or near the limit of light diffraction has resulted in the identification of numerous cell biological phenomena ^2– 7^.

For the purposes of conducting experimental imaging of cell biological specimens, the resolution limitations of light microscopy are somewhat balanced by the advantages inherent in the light microscopic technique. For example, it is possible to exceed the light diffraction limit and thereby achieve super-resolution images by using techniques such as atomic force microscopy, scanning tunnel microscopy ^8^, shear force microscopy, or other common types of scanning probe microscopy (SPM) ^9^. In addition, tried and true techniques such as electron microscopy (EM) generate extremely high-resolution images (sub-nanometer resolution), yet each of these imaging modalities manages to overcome the diffraction limit and achieve sub-diffraction limit resolution images by avoiding the use of visible light waves altogether. Additionally, the use of SPM and EM imaging methodologies for biological investigations often involves complicated and expensive sample preparation procedures. More importantly, because SPM and EM methods require physical “scanning” of the biological sample or require a fixed (non-living) sample, these super-resolution techniques have extremely low temporal resolution and do not allow imaging of the coordinated and dynamic movements of organelles, proteins, or other biological molecules located within subcellular processes. Thus, as experimental microscopists continue to use diffraction-limited light microscopy to piece together the finer details of the microscale architecture and dynamics of the cell, the need to break the light diffraction limit has become a focal point for biologists interested in defining the nanoscale organization of the cell with coordinately high spatial and temporal resolution.

This article reviews recent advances in super-resolution microscopy techniques as applied to microscopic imaging of subcellular processes. Ten to fifteen years ago, the concepts that underlie many of these super-resolution techniques were in place and were actively being tested, often with moderate to significant effect in increasing the resolution of biological samples and breaking the diffraction limit described by Abbe nearly 150 years before. In more recent years, super-resolution microscopy has continued down this path, heightening our ability to discern the biological details of primarily fluorescent molecules to single-nanometer resolution. Here, we discuss a variety of super-resolution imaging techniques first by describing the theoretical concepts that underlie the imaging methodology and then by providing examples of how some of these technologies have been applied to study subcellular biological processes. What is clear from the literature is that there has been an explosion of new imaging methods. As a result, we were forced to focus on a subset of the available super-resolution imaging approaches, and we apologize to the authors of the many optical techniques not discussed in this review.

## Stimulated emission-depletion microscopy

**Figure 1.**
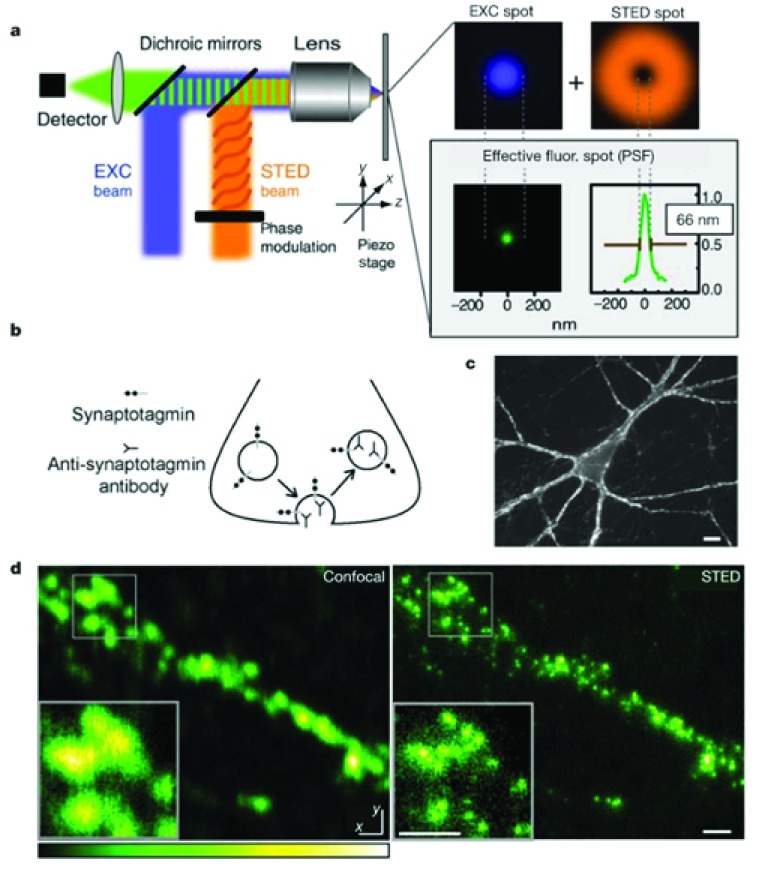
Stimulated emission-depletion (STED) microscopy resolves synaptic vesicles in individual boutons of primary cultured hippocampal neurons. ( **a**) Principles of operation. While the blue excitation (EXC) beam is focused to a diffraction-limited excitation spot, shown in the adjacent panel in blue, the orange STED beam is able to de-excite molecules. The STED beam is phase-modulated to form the focal dounut shown in the top right panel. Superimposition of the two focal spots confines the area in which fluorescence is possible to the dounut center, yielding the effective fluorescent spot of subdiffraction size shown in green in the lower panel. All spots represent measured data and are drawn to scale. The profile of the green effective fluorescent spot has a full width at half maximum of 66 nm as well as a sharp peak. The green spot shows an 11-fold reduction in focal area beyond the excitation diffraction value (compare with blue spot). ( **b**) Mechanism of synaptic labeling. Synaptic vesicles exocytose, allowing their lumenal synaptotagmin domains to bind anti-synaptotagmin antibodies. These antibodies are internalized upon endocytosis. ( **c**) Typical image of a neuron labeled with an anti-synaptotagmin antibody, fixed, permeabilized, and visualized by using Atto532-labeled secondary antibodies. Fluorescent puncta represent labeled synaptic nerve terminals. Scale bar = 10 mm. ( **d**) Comparison of confocal (left) and STED (right) counterpart images of a labeled preparation reveals a marked increase in resolution by STED. Scale bar = 500 nm. PSF, point spread function. Taken from 11.

The super-resolution microscopic technique termed stimulated emission-depletion (STED) was first described as a far-field imaging technique capable of producing 3D images with 35 nm resolution ^10^. STED microscopy operates by using two laser beams to illuminate the specimen. The first laser beam is an excitation laser pulse that is immediately followed (<1 ps) by a second red-shifted laser that illuminates the sample with a donut-shaped pattern. The STED approach is based on the theory that the full width at half maximum (FWHM) of the point spread function (PSF) can be reduced by the second laser beam that effectively takes a subpopulation of excited fluorescent molecules (from the first beam) and returns them to the ground state before they can spontaneously fluoresce. To achieve this, the STED laser intensity must be powerful enough that the stimulated emission can outcompete the fluorescence emission. Because the STED beam generates a non-linear depletion, the center of the beam has zero intensity while the periphery of the beam has high intensity (the donut-shaped pattern; ^11^; Figure 1). Thus, the excited fluorescent molecules in the periphery of the beam are turned “off” while those in the center remain “on”. The outcome is a narrowed PSF with an achieved FWHM approaching a range of 20 to 60 nm. The STED technique has been used to generate super-resolution images revealing that synaptotagmin remains clustered after synaptic vesicle exocytosis ( Figure 1).

In addition to the ability of STED to capture spatial and temporal super-resolution images, multicolor STED can investigate several different molecules simultaneously ^12^. Live-cell investigations using STED microscopy have been performed to investigate the dynamics of the syntaxin-1A protein with presynaptic vesicles ^13^ to define the super-resolution map of the ciliary transition zone ^14^ and to measure vesicular motility in living *Drosophila* ^15^. Recent studies have used STED microscopy to investigate the subcellular dynamics of the Bcl-2 family of mitochondrial proteins, Bax and Bak. This study found that when cells enter apoptosis, activated Bax molecules form large and compact clusters that assemble with Bak into ring-like structures in the mitochondrial outer membrane. These assemblies then contribute to the formation of large pores and suggest a mechanism for outer membrane permeabilization by Bax ^16^.

Achieving optimal super-resolution microscopic images depends largely on choosing the best tool(s) for the job. As with each super-resolution imaging approach, advantages of the STED technique are coupled with disadvantages. For biological imaging applications, the implementation of STED microscopy requires the use of STED probes that are specifically targeted to the protein of interest. Additionally, the fluorescent properties of the probes must be carefully evaluated because the resolution of STED relies on the characteristic saturation intensity and photostability of the fluorophore ^17, 18^. As a result, a relatively small number of fluorescent proteins and organic dyes are useful for STED imaging. Commonly used approaches for fluorescent labeling of proteins in live-cell applications, such as the green fluorescent protein, can be used in some cases for STED imaging; however, the brightness and quantum yield are often limiting, and there are few STED options for genetically encodable fluorescent labels in the far-red region, thus reducing the number of colors that can be imaged within the same specimen.

## Structured illumination microscopy

**Figure 2.**
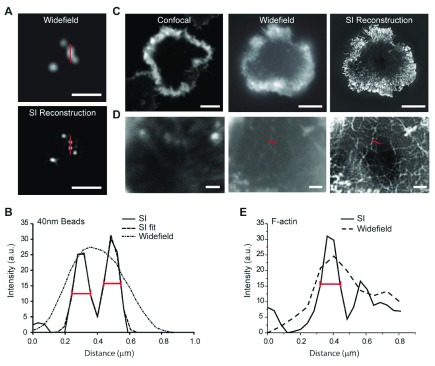
Structured illumination (SI) microscopy permits super-resolution imaging of F-actin at the natural killer cell synapse. ( **a**) Widefield (top) and SI images (bottom) of 40 nm beads. Bar = 1 μm. ( **b**) Plot profiles from the region marked by red bars in ( **a**) show that two beads approximately 200 nm apart can be resolved only following SI reconstruction. Fitting of the curve with two Gaussians gives full widths at half maximum (FWHMs) (red bars on the graph) of 99 and 105 nm for each bead, respectively. ( **c**) Confocal (left), widefield (middle), and SI image (right) of human primary natural killer cells activated on a surface coated with anti-NKG2D monoclonal antibody. Bars = 5 μm. ( **d**) Regions at the center of the synapses in ( **c**) are enlarged to demonstrate the increased level of detail in cortical F-actin structure when SI microscopy is used. Bars = 1 μm. ( **e**) Plot profiles to directly compare the widefield and SI reconstructed image for the region indicated by the red bar shown in ( **d**). Estimation of the FWHM (red bar on the graph) from the Gaussian fit of the SI data gives a resolution of approximately 115 nm. a.u., arbitrary units. Taken from 19.

Structured illumination microscopy (SIM) uses sample illumination with spatially structured excitation light of a known orientation. This approach makes normally inaccessible high-resolution information visible in the observed image. A series of images is reciprocally processed by using a Fourier transformation of the structured excitation image in order to generate a reconstruction with improved resolution. Preliminary experiments have confirmed the validity of the physical principle and the capability of this imaging methodology by using both test objects and complex biological structures to demonstrate superior effective resolution compared with conventional and confocal microscopes ( Figure 2) ^19– 21^. Performed in its linear form, SIM allows a doubling of the lateral resolution of a biological sample ^21^. Non-linear forms of SIM, for example, saturated pattern excitation microscopy and saturated SIM, couple linear SIM with ground-state depletion techniques resulting in lateral resolution of objects as small as 50 nm ^22^.

When the concept of structured illumination was first applied to imaging of biological samples ^21^, the approach used two superimposed line patterns of known geometry, which mathematically results in the multiplication of the line patterns with the fluorescently labeled sample structure, with the product containing moiré fringes. The method of imaging subcellular processes can be combined with Fourier transforms by overlapping two different spatial frequencies from multiple directions to obtain finer spatial frequencies emitted by the specimen. By using structured illumination to acquire a sequence of images, each with different orientation and phase of the pattern, information is recovered from twice the area of what is observable using confocal microscopy, and a resulting doubling of the resolution, including lateral resolutions in the range of 100 nm and axial resolutions approaching 300 nm.

Recent advances using structured illumination technology have combined non-fluorescent Raman imaging with SIM technology to increase the spatial resolution of both biological and inorganic chemical mapping ^23^. Additionally, investigations of the subcellular structures termed focal adherens junctions (FAJs) have used SIM to identify that tension-sensitive focal adhesion protein members display distinct localization patterns within FAJs that may function as a force-sensitive module capable of regulating specific aspects of junction dynamics ^24^.

Other experimental investigations have incorporated 3D two-color SIM datasets of living cells to visualize super-resolution time-lapse dynamics of mitochondria, vesicles, and the actin cytoskeleton on the order of 8.5 seconds per two-color image (4 seconds for single color) ^25^. As with all existing super-resolution microscopic techniques, efforts toward enhancing SIM have focused on both increased spatial resolution and enhanced temporal resolution needed to visualize dynamics of subcellular components. Examples of SIM-associated advances in temporal resolution include two-photon point-based scanning methods, which have the ability to acquire super-resolution images approximately one image per second ^26, 27^, in addition to the acquisition of 3D super-resolution images in thick, semi-transparent biological specimens ^27, 28^. Yet another SIM modality, termed “instant SIM” (iSIM), has achieved the most temporally resolved super-resolution imaging. The iSIM approach is optimized for live-cell imaging of 2D or 3D biological samples and is capable of achieving 145 nm lateral and 320 nm axial resolution at frame rates faster than 100 frames per second ^29– 31^.

## Single-molecule imaging modalities

Some of the first applications for single-molecule detection combined optical force traps with single-molecule fluorescence techniques to characterize nucleotide dynamics and force production of myosin motors ^32– 34^. Subsequently, a theoretical concept underlying single-molecule imaging was demonstrated in 2003 and was termed fluorescence imaging with one-nanometer accuracy (FIONA) ^35^. This methodology was used to perform single-molecule measurements of the myosin V molecular motor protein and showed in exquisite detail that myosin V walks along actin filaments in a hand-over-hand mechanism. Perhaps of critical importance to the future of super-resolution microscopy, the FIONA technique showed how fluorescent molecules could be localized with sub-diffraction precision by curve-fitting fluorescence emission data (the PSF) to determine the mean value of the emission distribution and the corresponding standard deviation of the distribution. The PSF is defined by four variables that also define the diffraction limit of resolving two microscopic points or molecules: (1) the number of collected photons (highly influenced by the numerical aperture of the objective lens), (2) the pixel size of the detector, (3) the standard deviation of the background noise plus detector noise, and (4) the standard deviation of the signal (this determines the precision of the PSF, also known as the FWHM).

Limitations of FIONA have since been overcome by modification of this imaging approach. For example, FIONA can measure a single molecular domain over time but cannot resolve multiple fluorescence emitters in close proximity. The commonality of multiple emitters led to further developments in the theoretical technique in order to eliminate or at least to reduce this obstacle. Photo-bleaching of a subset of fluorescent emitters was one methodology capable of reducing the multiple-emitter problem. Soon, numerous adaptations of FIONA were employed to answer important cell biological questions that required sub-diffraction resolution of single molecules, including nanometer-localized multiple single-molecule fluorescence microscopy ^36^, single-molecule high-resolution imaging with photo-bleaching ^37^, single-molecule high-resolution co-localization microscopy ^38^, stochastic optical reconstruction microscopy (STORM) ^39^, and photo-activated localization microscopy (PALM) ^40^. In both of these single-molecule imaging modalities, the addition of turning off the fluorescent probe via photo-switching or photo-bleaching was the key underlying principle that made these techniques capable of distinguishing multiple fluorescent emitters within a biological sample.

### Photo-activated light microscopy

PALM imaging took the FIONA concept of curve-fitting a PSF to generate a super-resolution image and combined it with photo-activatable or photo-switchable (PA) fluorescent molecules ^40^. This approach is ideal for generating high signal-to-noise ratios because the sample is essentially “dark” until the PA wavelength of light is applied to the sample. Additionally, the PA approach permits controlled levels of photo-activation (by modifying activation laser light intensity or exposure times) of whole cells or within sub-cellular processes (using an imaging system capable of illuminating user-defined regions of interest).

PALM imaging uses a repetitive process in which a subset of the PA fluorophores within a biological sample is first excited, an image is acquired, and then the excited fluorophores are turned off by either photo-switching or photo-bleaching the fluorescent probe(s). This approach allows a small number of fluorophores to emit photons with each round of imaging. After repetition of this process potentially hundreds to thousands of times on a single sample, the distribution and localization intensity of each acquired PSF can be assigned computationally to generate the super-resolution image ^40^. The ultimate resolution achievable in PALM is dependent on the localization precision (how well the center of each PSF can be determined) and the density of available PA molecules. Thus, the four contributing PSF variables (described above) apply to PALM imaging, as well as the density of the PA molecules. Both 2D and 3D PALM techniques are complemented by the use of total internal reflection fluorescence (TIRF) microscopy ^41, 42^. Although TIRF is not theoretically required to perform PALM imaging, the advantage of TIRF is the use of an evanescent wave of illumination that generates a relatively narrow depth of field (~200 nm) for imaging. Thus, TIRF microscopy inherently limits the amount of out-of-focus light that reaches the objective lens and thereby increases the resolution of the resulting image. The high signal-to-noise ratio gained by TIRF microscopy is excellent for imaging focal adhesions or membrane-associated proteins that are within the 200 nm evanescent illumination plane, but it also highlights the limitations of the TIRF approach to a narrow depth of imaging field.

At the time it was first developed, one inherent limitation of the PALM technique was that it required the biological sample to be fixed (non-living) and thereby removed the possibility of live-imaging the dynamics of the system. Adaptations of the original PALM technique have since allowed live-cell investigations to obtain measurements of nanoscale dynamics of adhesion complexes ^43^, T-cell signaling ^44^, and a number of membrane-bound protein investigations ^45– 47^. Still, the major limitation of PALM imaging is low temporal resolution because the need for spatial distinction of photo-activated molecules, which by its nature results in low quantum yield, requires increased acquisition time to overcome this obstacle. Therefore, live-cell PALM imaging provides substantially increased spatial resolution but suffers from low temporal resolution and therefore has not been used to image temporally dynamic subcellular events. However, new innovations of PALM have made breakthroughs that significantly reduce the time necessary to acquire PALM images and provide hope for continued enhancements in temporal resolution using this imaging modality ^48^.

PALM imaging of subcellular processes has been used to conduct 2D analysis of focal adhesion complexes ^43^ and, using a PALM modification termed “interferometric” PALM (iPALM), to investigate the 3D structure of focal adhesion complexes ^49, 50^. Two-dimensional studies of focal complexes using a single- or dual-color PALM approach have shown the stratified organization of the focal adhesion molecules vinculin and alpha-actinin in super resolution. These results were compared with images of the same fluorescent molecules within the same adhesion structures to highlight the enhanced resolution of the PALM technique.

**Figure 3.**
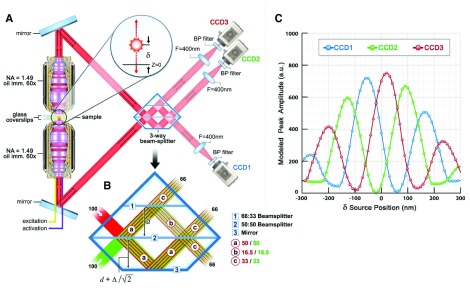
Schematics and operating principle of multiphase interferometric microscope illustrating how z-position is resolved. ( **a**, **b**) Schematic of the single-photon multiphase fluorescence interferometer. A point source with z-position δ emits a single photon both upwards and downwards. These two beams (color-coded as red and green in ( **b**) interfere in a special three-way beam splitter. ( **c**) The self-interfered photon propagates to the three color-coded charge-coupled device (CCD) cameras with amplitudes that oscillate 120° out of phase as indicated. a.u., arbitrary units; BP, band pass; NA, numerical aperture. Taken from 49.

**Figure 4.**
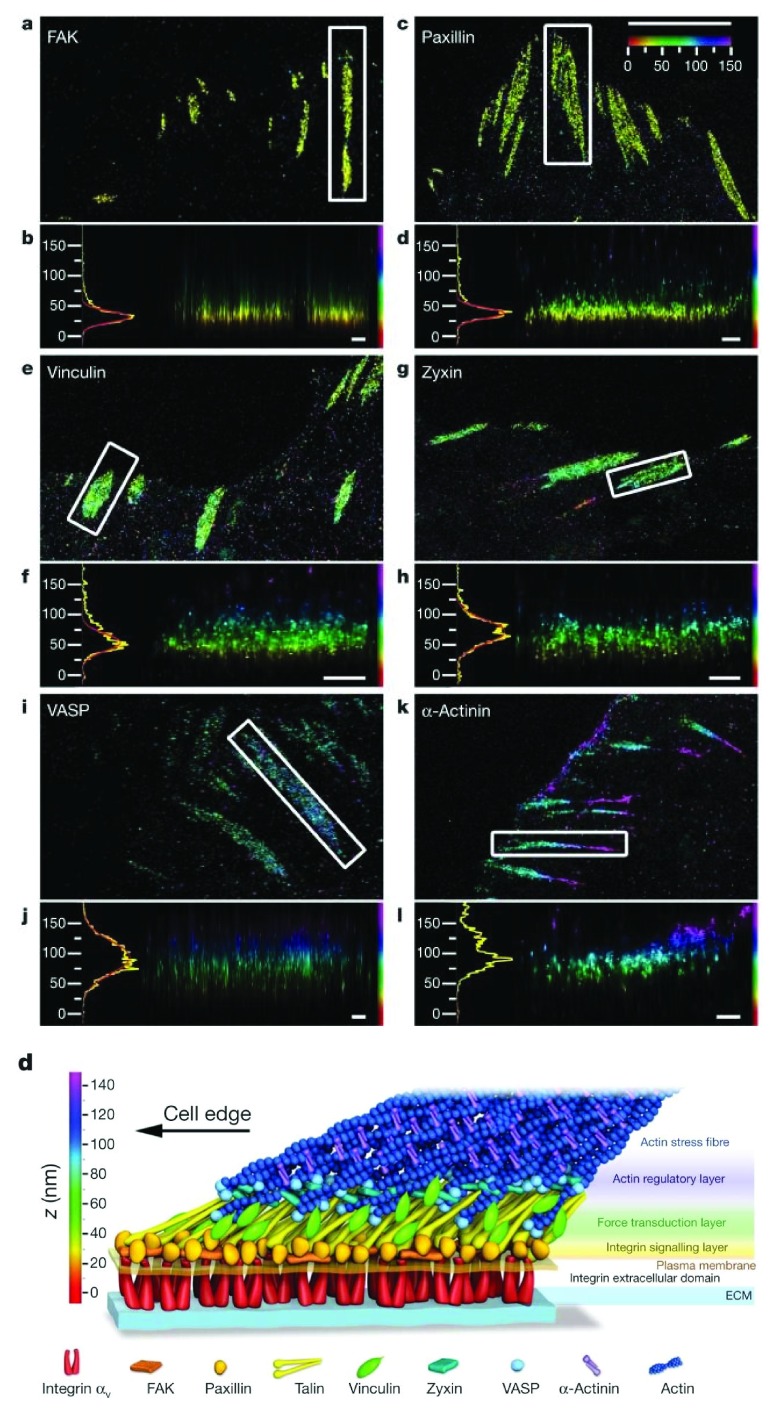
Protein stratification of the focal adhesion core. Top view and side view “interferometric” photo-activated localization microscopy (iPALM) images of focal adhesions (white boxes, top-view panels) and corresponding *z* histograms and fits. ( **a**, **b**) FAK. ( **c**, **d**) Paxillin. ( **e**, **f**) Vinculin. ( **g**, **h**) Zyxin. ( **i**, **j**) VASP. ( **k**, **l**) Alpha-actinin. The vertical distribution of α-actinin is non-Gaussian, so the focal adhesion peak fit is not shown. Paxillin and α-actinin shown are C-terminal photo-activatable fluorescent protein (PA-FP)-tagged. Colors: vertical ( *z*) coordinate relative to the substrate ( *z* = 0 nm, red). (d, bottom panel). Schematic model of focal adhesion molecular architecture depicts experimentally determined protein positions. Note that the model does not depict protein stoichiometry. Scale bars = 5 μm (a, c, e, g, i, k) and 500 nm (b, d, f, h, j, l). ECM, extracellular matrix. Taken from 50.

To apply the PALM technique to investigations of the 3D organization of subcellular structures, investigators developed a modification termed iPALM, which is the combination of PALM with single-photon, simultaneous multiphase interferometry that provides sub-20 nm 3D protein localization with optimal molecular specificity ^49^. Using the well-defined principle of self-interference of a single-photon source, iPALM orients a biological sample containing PA-labeled molecules at the focal plane of two opposed objective lenses in order to allow for the precise determination of each molecule’s axial position ( Figure 3). Upon illumination, a fluorescent photon simultaneously enters both the upper and lower objectives and the difference in path lengths of the upper and lower beams directly depends on the axial position of the source. Additionally, self-interference in the three-way beam splitter results in an axial-dependent modulation of the relative intensities of the three output beams. This concept enabled the 3D position of the source molecule to be determined from the relative amplitudes of the source images from the three cameras ( Figure 3). This technology was most prominently applied in a study that mapped the nanoscale organization of focal adhesion proteins ( Figure 4) ^50^.

### Stochastic optical reconstruction microscopy

In the same way that PALM imaging uses stochastic activation of PA fluorescent probes, the fundamental principle behind STORM is that the activated state of a photo-switchable molecule must lead to the consecutive emission of sufficient photons to enable precise localization before it enters a dark state or becomes deactivated by photo-bleaching. Additionally, the sparsely activated fluorescent molecules must be separated by a distance that exceeds the Abbe diffraction limit (approximately 250 nm) to enable the parallel recording of many individual emitters, each having a distinct set of coordinates in the lateral image plane.

**Figure 5.**
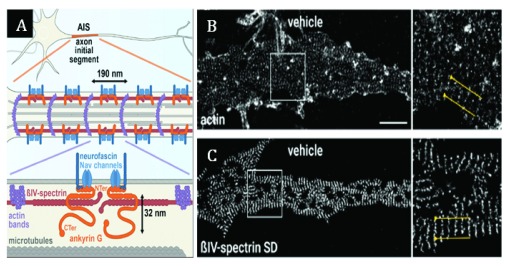
Schematic graphical representation of the axon initial segment constructed from stochastic optical reconstruction microscopy (STORM) imaging data of βIV spectrin, ankyrin G, actin, and microtubules. ( **a**) The schematic shows that head-to-head βIV-spectrins connect the actin rings to form a periodic sub-membrane complex while ankyrin G C-terminal tails extend 32 nm below the sub-membrane complex to allow possible interactions with the microtubule cytoskeleton that are important for establishing and maintaining axonal identity. ( **b**, **c**) STORM images of neurons treated with vehicle (dimethyl sulfoxide 0.1%; 1 hour; **b**, **c**) and then fixed and labeled for actin ( **b**) or βIV-spectrin specific domain (SD) ( **c**). Scale bar = 2 μm. Taken and modified from 55.

Biological applications of the STORM imaging methodology ( Figure 5) have revealed that axonal actin is organized as ring-like structures spaced every 180 to 190 nm and that this periodic arrangement of actin is linked to the organization of spectrin and ankyrin proteins within a region of the neuron termed the axon initial segment (AIS) ^51^. The physiological significance of this organization in neurons is proposed to somehow operate as a transport “scaffold” or “filter” that functions to specifically distinguish the AIS from other regions of the neuron to maintain the identity of the axon ^52– 54^. The fine molecular details of the AIS scaffold were recently identified by using multicolor 2D- and 3D-STORM of endogenous epitopes to demonstrate that the periodic organization is composed of longitudinal head-to-head βIV-spectrin subunits that work to connect actin-rich bands along the AIS ^55^. Furthermore, this study used STORM imaging to identify that ankyrin G scaffold protein is associated with the AIS membrane, where it extends approximately 35 nm into the membrane-adjacent cytoplasm in order to possibly facilitate an interaction with peripheral microtubules and thereby regulate vesicular entry into the axon ( Figure 5) ^55^. These data suggest that the organization of AIS components is necessary for defining axon integrity by establishing a subcellular gateway to the axon.

STORM imaging has also been used to discern fine details of the microtubule-organizing center, including the pericentriolar material (PCM), which has remained elusive in its composition for decades ^56^. This study combined SIM and STORM imaging to attempt to identify the organization of the PCM in *Drosophila*. STORM imaging revealed that the Pericentrin-like protein (Plp) is distributed in distinct molecular clusters around the centriole and forms molecular fibrils extending into the PCM matrix, comprising a scaffold similar to spokes of a wheel. The combined SIM/STORM study demonstrated that the PCM is composed of two distinct structural layers that provide separate functionality. Moreover, the investigators discovered that Plp plays an important role in PCM organization by forming a molecular scaffold for the PCM matrix ^56^. The combination of these two super-resolution methodologies was necessary because of the limitations of STORM sample preparation ^57^. The ability to overcome these limitations or to invent new feasible methods of sample preparation will likely determine the utility of STORM imaging of nanoscopic cellular structures.

## Imaging live specimens

During traditional epifluorescence, confocal, and many of the super-resolution microscopy techniques, the specimen is illuminated with an extensive beam that travels through the whole sample. The problem with this light path is that the two cones of light excite the out-of-focus components of the sample. While techniques such as spinning disk confocal microscopy that allow for full-field confocal imaging have helped with live-specimen imaging, any excess light as found in these techniques is damaging to the sample, releasing free oxygen radicals, bleaching the fluorescent probes, and even heating the specimen. This often will cause artifacts such as cell retraction or shrinkage, and may result in cell death. Many of the techniques described above are still not very useful for live-cell imaging, often requiring that the cells be fixed. Better techniques are being developed that provide the ability to image many frames over short durations for the examination of dynamic cellular events. These four-dimensional (4D) techniques can also be valuable for obtaining more subtle changes occurring over many hours or even days.

## Light sheet fluorescence microscopy

**Figure 6.**
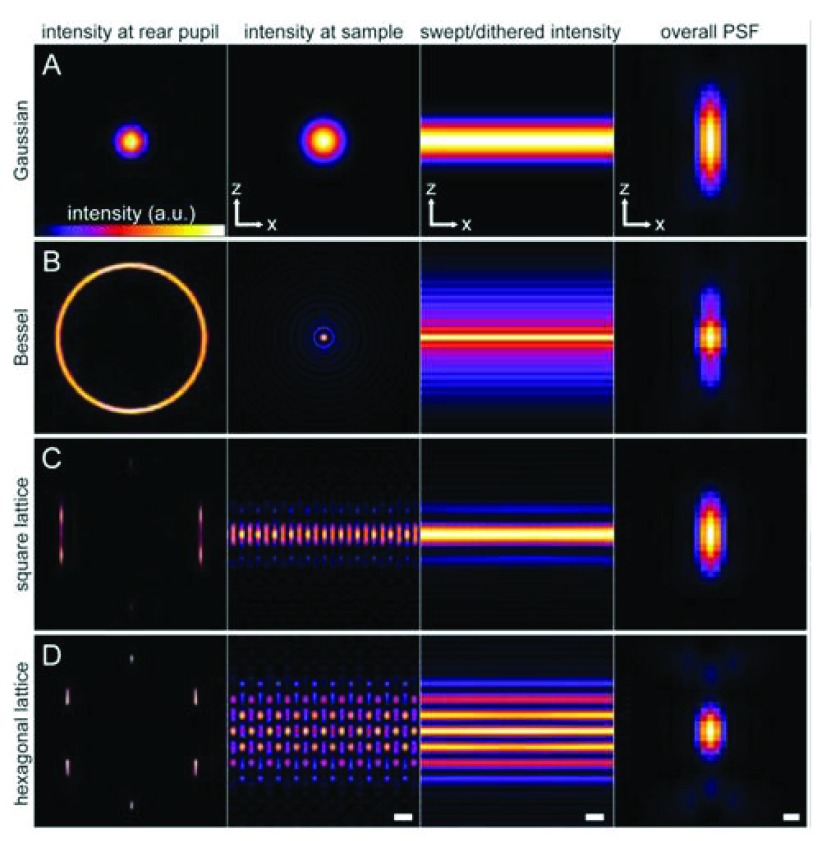
Methods of light sheet microscopy. ( **a**) The traditional approach, in which a Gaussian beam is swept across a plane to create the light sheet. ( **b**) A Bessel beam of comparable length produces a swept sheet with a much narrower core but flanked by sidebands arising from concentric side lobes of the beam. ( **c**, **d**) Bound optical lattices create periodic patterns of high-modulation depth across the plane, greatly reducing the peak intensity and the photo-toxicity in live-cell imaging. The square lattice in ( **c**) optimizes the confinement of the excitation to the central plane, and the hexagonal lattice in ( **d**) optimizes the axial resolution as defined by the overall point spread function (PSF) of the microscope. The columns in ( **a** to **d**) show the intensity pattern at the rear pupil plane of the excitation objective; the cross-sectional intensity of the pattern in the *xz* plane at the focus of the excitation objective (scale bar = 1.0 μm); the cross-sectional intensity of the light sheet created by dithering the focal pattern along the *x* axis (scale bar = 1.0 μm); and the *xz* cross-section of the overall PSF of the microscope (scale bar = 200 nm). Taken from 69. Abbreviations:AU; arbitrary units.

To help address this problem of specimen photo-damage, several techniques have been developed in which only the imaging plane of focus is illuminated with a “sheet” of light. Planar illumination was first proposed by the 1925 chemistry Nobel Prize winner Richard Adolf Zsigmondy along with German physicist Heinrich Siedentopf in 1902 ^58^. The technique was revitalized in the late 1990s when microscopists realized its potential for experimental use as new fixed and live-cell fluorescent probes came on line for imaging subcellular organelles and molecules. In 2004, Huisken *et al*. published an article demonstrating the viability of selective plane illumination microscopy (SPIM), which is synonymous with light sheet fluorescence microscopy (LSFM) ^59^. These systems typically have an imaging objective and an orthogonal cylindrical lens or excitation objective that excites the specimen with a thin sheet of light. Because optical sectioning is achieved by the excitation light sheet, an advantage of SPIM is that the entire focal plane is imaged simultaneously ( Figure 6a). SPIM was found to be useful for imaging large, multicellular specimens, but the benefits of using SPIM for single-cell microscopy are limited. This is because of the inherent nature of the illumination and the divergence of the beam. The illumination can become thinner in only a relatively small region, typically in the z direction. The thinner the sheet is, the narrower it becomes in x or y, and the faster it diverges (fattens) elsewhere in the sample. This does not allow the sheet to get much flatter than a cell before it becomes a focused point of light. Nevertheless, this configuration permits fast imaging of an entire plane that can be imaged with sensitive complementary metal-oxide-semiconductor or EM charge-coupled device cameras. Whole volume collection can be acquired either by scanning the sample through a stationary objective or by moving the objective synchronously to scan a stationary sample. This has been useful for imaging large specimens.

## Bessel beam microscopy

The development of Bessel beam microscopy has provided a solution for imaging smaller features and cells by creating a beam that can illuminate the sample in a planar manner ^60, 61^. Bessel beams are a class of non-diffracting beams that will maintain a relatively tight and unabberated focus over a prolonged distance of approximately 50 μm ^62, 63^. The Bessel beam enters the lens only on the periphery of the lens, creating a funnel of light with a flat focus point ( Figure 6b). Despite the flat focus of the Bessel beam, imaging studies using this technique revealed problems with out-of-focus excitation. To overcome this obstacle, two-photon excitation has been introduced to the Bessel beam system ^63– 66^. Two-photon excitation diminishes by the square of the excitation intensity, and the outer-lying lobes of the Bessel beam become less relevant ^67^. This approach comes with all the typical limitations of two-photon microscopy, including the cost, but does allow the deeper imaging into tissue specimens.

Additional functionalities were added to the Bessel beam approach by creating a Bessel beam super-resolution SIM, which adds the principles of 3D super-resolution SIM (3D SR-SIM) ^65, 68^. The Bessel beam SR-SIM has an axial resolution that is approximately two times that of confocal techniques and roughly similar to that of widefield SR-SIM.

## Lattice light sheet microscopy

More recently, the Betzig lab has engineered a Bessel beam microscope with multiple beams that allow the creation of a lattice light sheet microscope ^69^. Optical lattices are periodic interference patterns in two or three dimensions created by the coherent superposition of a finite number of plane waves travelling in certain well-defined directions. An ideal 2D lattice is non-diffracting in the sense that it propagates indefinitely in a direction y without changing its cross-sectional profile, which extends infinitely in x and z. This is accomplished by confining the illumination at the rear pupil plane of the excitation objective to points on an infinitesimally thin ring. This allows one to image a specimen with the same total flux as measured with individual spots in confocal microscopy, but that flux is dispersed among the multiple beams and the cell is exposed to reduced amounts of excitation illumination ( Figure 6d) ^69^. This lattice light sheet microscopy (LLSM) had clear advantages over the single Bessel beam microscope, including an approximately 75% reduction in the total radiation delivered and the ability to image approximately three times faster for patterns of a similar period. This approach also allows imaging with finer patterns that require fewer steps per plane, resulting in a 3D volume acquisition that is several times faster than with the Bessel approach ^69^. Chen *et al*. ^69^ (2014) demonstrated the advantages of the LLSM by imaging 20 different specimens spanning four orders of magnitude in space and time, including the binding kinetics of individual transcription factors, 3D super-resolution PALM of nuclear lamins, dynamic organelle rearrangements and 3D tracking of microtubule plus ends during mitosis, neutrophil motility in a collagen mesh, and subcellular protein localization and dynamics during embryogenesis in *Caenorhabditis elegans* and *Drosophila melanogaster*. As an example of the minimal amount of phototoxicity elicited on a specimen, a single ciliated *Tetrahymena thermophila* cell was imaged continuously for over 7 minutes, and over 200,000 frames were acquired. In 2D mode, 18,000 frames were acquired at 3-millisecond intervals highlighting the beating of cilia and the movement of subcellular organelles. In addition, full 3D volumes of the microtubule cytoskeleton in a *Dictyostelium discodeum* cell were acquired for 1,430 frames, and volumes were taken every 2 seconds, and there was no noticeable photobleaching or phototoxicity ^69^.

## Inverted selective plane illumination microscope

One limitation of the above LLSM approaches is the arrangement of the sample holder, which limits the type of samples you can observe and often makes interacting with the sample with micromanipulators or even perfusion difficult. An easier sample mounting approach was developed with the inverted selective plane illumination microscopy (iSPIM), which uses a standard inverted microscope platform ^70^. For excitation, a laser passes through a mask and a mirror directs it into a 45° oriented excitation objective mounted on an anchor pillar which directs the excitation light sheet toward the specimen. The fluorescence is collected by a second 45° oriented objective. A second camera can be positioned under the inverted microscope platform objectives for focus-finding and transmitted light purposes. This type of imaging was used to collect volumes every 2 seconds for a 14-hour period of embryogenesis (collecting approximately 25,000 volumes) with no detectable phototoxicity in a *C. elegans* nematode. They also performed two-color imaging that allowed them to perform lineage tracing while also visualizing neurodevelopmental dynamics ^70^. Other groups have used similar LLSM techniques for 4D imaging in zebrafish embryos ^71^ and in the fruit fly *Drosophila melanogaster* ^72, 73^. The iSPIM has also been modified to capture a second specimen view by alternating excitation and detection between the two objectives. The resulting “diSPIM” provides isotropic spatial resolution (down to 330 nm) at high speed (200 images per second, 0.5 seconds for a dual-view 50-plane volume) and has been used to successfully track microtubule tips in three dimensions in living cells as well as improve the imaging of nuclear dynamics during *C. elegans* embryogenesis ^74^. The Shroff group also has provided step-by-step directions for the construction of a diSPIM entirely from commercially available parts ^75^. This design is compatible with fiber-coupled laser excitation. Such fiber-based excitation facilitates alignment, making the device compatible with a broad array of commercial laser excitation sources. Several other groups have provided instructions to build LLSM systems, making it possible for biologists to build them from commercially available microscope equipment ^76, 77^.

## Spherical-aberration-assisted extended depth of field

A brand-new technique called spherical-aberration-assisted extended depth-of-field light sheet microscopy (SPED-LSM) was recently published ^78^. This method turns spherical aberration into an advantage by combining the large volumetric field of view of an extended depth of field with the optical sectioning of light sheet microscopy. The problem with the use of spherical aberration is it reduces the contrast and resolution in both the focal plane and optical axis. In part, the authors correct for this through the use of deconvolution. The SPED-LSM technique used galvanometer scanners, which eliminated the need to physically scan the detection objective. SPED enabled scanning of thousands of volumes per second, limited only by camera acquisition rate. The authors demonstrate capabilities of SPED microscopy by performing fast subcellular resolution imaging of CLARITY ^79^ (a technique that produces structurally intact yet optically transparent tissue) mouse brains and cellular-resolution volumetric Ca ^2+^ imaging of entire zebrafish nervous systems. It is easy to imagine integrating a SPED system with complementary optics for optogenetics so one might simultaneously record and control neural activity across portions and maybe even the entire vertebrate nervous system. This type of microscopy will have a lot of potential as camera speeds increase.

## Concluding remarks

This is an exciting time to be a cell biologist. However, with so many new types of super-resolution microscopy techniques now available, it becomes a significant challenge to determine which microscopes are useful for your studies. One must consider what type of spatial and temporal resolution is needed to answer the questions at hand. The cell type, the type of fluorophores being used, the time between acquisitions, and the duration of the experiment all can have a significant influence on the type of microscopy that can or should be performed. With fixed specimens, one can generally illuminate the samples with much more excitation light without worrying about generating artifacts. For live-specimen microscopy, sometimes going faster and seeing smaller details provides useful information; other times it does not. One still must be careful not to oversample and damage the specimen even with some of these very gentle LSFM techniques. One thing that is quickly becoming a problem is the handling of the raw data generated with some of these new 4D techniques. Data management can quickly become a burden since acquiring 4D data with many of these advanced techniques can quickly lead to the generation of terabytes of data that need to be organized, examined, and analyzed. Once interesting data are identified, the scientific community also needs to determine better ways to make all of the data accessible. So many exciting findings are just buried away on the hard drives of research laboratory computers and never see the light of day or the right set of eyes that might recognize an important discovery. The good news is that memory storage is becoming reasonably priced, maintaining your data in “the cloud” is easier, and journals are also allowing larger sized files so that the scientific community has the ability to visualize these exciting new techniques when they are published.
